# Gemcitabine and carfilzomib induced thrombotic microangiopathy: eculizumab as a life‐saving treatment

**DOI:** 10.1002/ccr3.1214

**Published:** 2017-10-09

**Authors:** Rahul Gosain, Amitoj Gill, Jacob Fuqua, Lesley H. Volz, Mika R. Kessans Knable, Ryan Bycroft, Sarah Seger, Rohit Gosain, Jorge A. Rios, Ju‐Hsien Chao

**Affiliations:** ^1^ Division of Hematology and Medical Oncology James Graham Brown Cancer Center University of Louisville School of Medicine Louisville Kentucky; ^2^ Department of Medicine University of Louisville School of Medicine Louisville Kentucky; ^3^ Department of Pharmacy James Graham Brown Cancer Center University of Louisville Louisville Kentucky; ^4^ Division of Hematology and Medical Oncology Roswell Park Cancer Institute Buffalo New York; ^5^ Division of Hematology and Medical Oncology The Mark H. Zangmeister Cancer Center Columbus Ohio; ^6^ Division of Hematology and Medical Oncology Texas Transplant Physician Group ‐ Methodist Physicians San Antonio Texas

**Keywords:** Carfilzomib, eculizumab, gemcitabine, hemolytic–uremic syndrome, thrombotic microangiopathy, thrombotic thrombocytopenia purpura

## Abstract

Drug‐induced aHUS is rare; however, early diagnosis is vital to reduce morbidity and mortality. With confirmation of the diagnosis, eculizumab appears to be a viable treatment option to suppress the pro‐inflammatory surge. Furthermore, adverse side effects of medications such as carfilzomib and gemcitabine should be considered in the appropriate settings.

## Background

Historically, the presentation of thrombotic microangiopathy (TMA) with acute renal insufficiency, thrombocytopenia, and microangiopathic hemolytic anemia was only associated with thrombotic thrombocytopenic purpura (TTP) or hemolytic–uremic syndrome (HUS). Advances in our understanding of the etiology of TMA have allowed the diseases to be further classified based on causation rather than clinical association. The known etiologies of TMA include infection‐induced– HUS, ADAMTS13 deficiency – TTP, disorders of complement regulation – complement‐associated hemolytic–uremic syndrome also known as atypical hemolytic–uremic syndrome (aHUS), defective cobalamine metabolism – newborn HUS, and Quinine‐induced 1. After one has excluded infection or ADAMTS13 deficiency, another life‐threatening differential diagnosis, aHUS as a possible cause of the patient's presentation, should be considered.

aHUS is defined as a non‐Shiga toxin, nonimmune TMA caused by uninhibited continuous activation of the alternative complement system with an overall incidence of 1–3 cases per 100,000 2, 3. It is estimated that less than half of aHUS cases are familial in origin, resulting from genetic or complement abnormalities, whereas other cases are sporadic in nature triggered by infection, malignancy, pregnancy, organ transplantation, or drugs 2. Although a rare entity, multiple mechanisms including acute immunologic reaction, direct toxic effect, acute dose‐related toxicity, or chronic and duration‐dependent toxicity have been proposed for drug‐induced aHUS 4.

Certain chemotherapy agents such as gemcitabine and mitomycin have a higher incidence of drug‐induced aHUS; however, new anticancer drugs including proteasome inhibitors have an increasing number of cases reported as well. Other classes of drugs commonly associated with this presentation are immunosuppressive agents, namely cyclosporine, tacrolimus, and sirolimus or antiplatelet agents including clopidogrel and ticlopidine 1, 2, 4, 5.

Gemcitabine is a known nucleoside analogue of cytarabine that was first synthesized in the early 1980s with intent to be used as an antiviral agent, but with in vitro testing, it appeared to be a promising anticancer drug 6. Gemcitabine was first approved in the United Kingdom in 1995 and then followed by US Food and Drug Administration (FDA) in 1996. It is commonly used in first‐ and second‐line treatment for non‐small‐cell lung, bladder, ovarian, pancreatic, and breast cancers 7. Thrombocytopenia and anemia from bone marrow suppression are common side effects of gemcitabine, but the first case of gemcitabine‐induced TMA was reported in 1994 during an ongoing phase II clinical trial for pancreatic adenocarcinoma 8. Fortunately, the incidence of gemcitabine‐induced hemolytic–uremic syndrome has been reported to be between 0.02% and 2.2% 8, 9.

With an increasing number of FDA‐approved drugs and indications, the total number of drug‐induced aHUS cases is on a rise. Carfilzomib, a selective proteasome inhibitor recently approved for relapsed and refractory multiple myeloma (MM), has also been associated with drug‐induced aHUS similar to its predecessor, bortezomib 10. The ubiquitin–proteasome pathway plays an important role in the cell cycle, and bortezomib was the first proteasome inhibitor to be widely used as part of MM therapy followed by a second‐generation proteasome inhibitor, carfilzomib. Hobeika L et al. reported the first case of carfilzomib‐induced renal thrombotic microangiopathy; however, there was no sign of microangiopathic hemolytic anemia 11. Recently, Lodhi A. et al. documented a case of drug‐induced aHUS associated with carfilzomib meeting all TMA criteria.

The treatment of TMA traditionally has been plasma exchange (PLEX) and supportive management; unfortunately, most reports state that PLEX has little to no effect on renal function, thrombocytopenia, or anemia 12. With better understanding of underlying pathophysiology for drug‐induced aHUS, early recognition is important, and we propose that eculizumab, a monoclonal antibody that binds to complement protein C5, in addition to supportive care, could be a viable treatment to shorten the course of this complement‐excited state and decrease recovery time in this patient population.

## Case Presentation

### Patient 1

**Figure 1:**
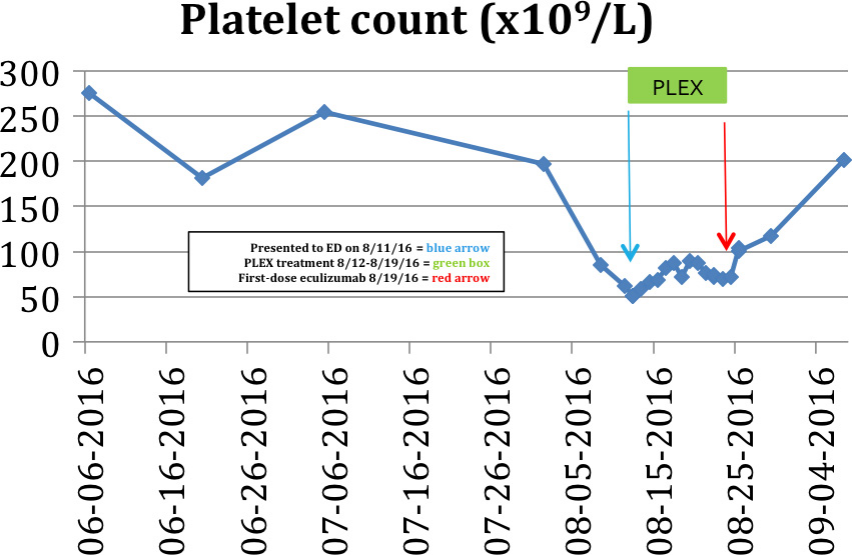
Patient 1 Platelet response to PLEX and eculizumab.

**Figure 2:**
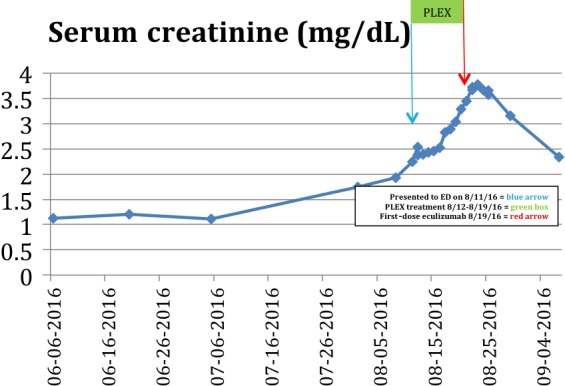
Patient 1 Serum Creatinine response to PLEX and eculizumab.

A 54 year old woman diagnosed with pancreatic adenocarcinoma, status postsurgical resection and on cycle 12 day 11 of palliative chemotherapy (gemcitabine and nab‐paclitaxel), presents to the hospital with ongoing headache. The patient was noted to have elevated blood pressure, elevated serum creatinine (Cr) at 2.45 mg/dL (from 1.10 mg/dL), hemoglobin (Hb) of 8.1 g/dL (from 10.5 g/dL), and platelet (Plt) count of 62 × 10^9^/L (from 215 × 10^9^/L). Further work‐up revealed LDH of 419 IU/L and haptoglobin of <10 mg/dL. Peripheral blood smear (PBS) was consistent with 3–5 schistocytes on each high‐power field (HPF). Given the patient's clinical and laboratory data, PLEX was initiated for a presumed diagnosis of TMA. The patient was maintained on PLEX for 7 days along with a nicardipine intravenous drip and four scheduled antihypertensive medications with minimal response in blood pressure, renal function, thrombocytopenia, and anemia. With a further decline in her Plt count, Hb, renal function (Cr of 3.54 mg/dL), and sufficient ADAMTS13 activity (79%), diagnosis of TTP was excluded and eculizumab was initiated. Forty‐eight hours after the first eculizumab dose (900 mg IV), the patient was weaned off nicardipine and her Plt count and Hb stabilized. Her serum creatinine continued to rise and peaked at 3.78 mg/dL 5 days after the first dose. The second dose of eculizumab (900 mg IV) was given 2 days earlier (on the 5th day) in order to saturate the C5 protein and anticipate a response sooner. The patient was discharged and seen in outpatient clinic, prior to her 3rd dose of 900 mg IV eculizumab. Patient was maintained on 900 mg IV eculizumab till week 4 (every week) followed by 1200 mg IV on 5th week every 2 weeks as per the approved dosing schedule. The patient remained on three antihypertensive medications and was noted to have normalization of platelet count, 201 × 10^9^/L (Fig. 1), increase in Hb, 9.6 g/dL, and a decrease in SCr, 2.15 mg/dL (Fig. 2).

### Patient 2

**Figure 3:**
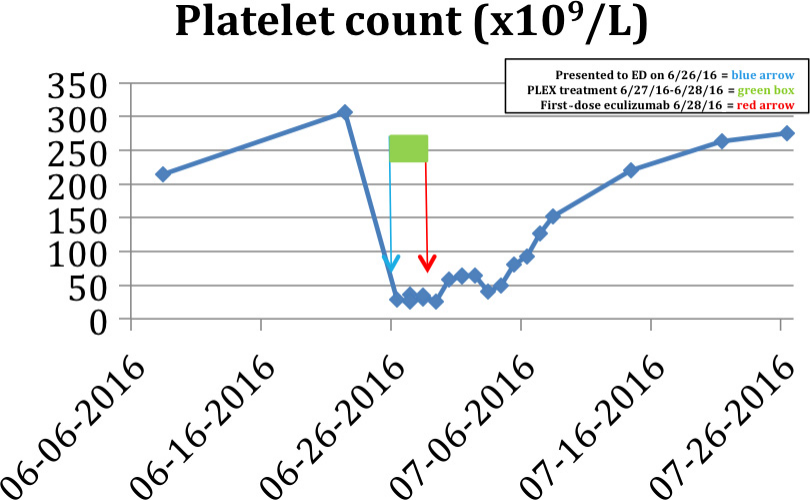
Patient 2 Platelet response to PLEX and eculizumab.

**Figure 4:**
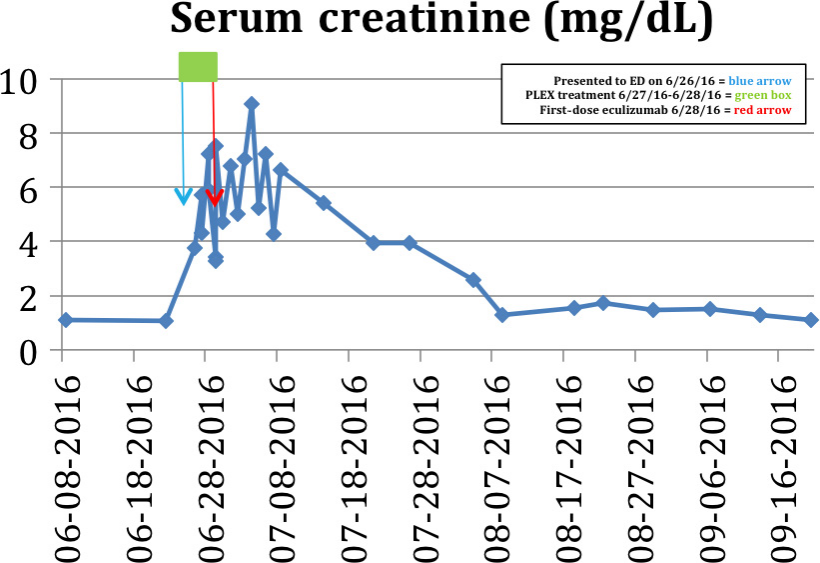
Patient 2 Serum creatinine response to PLEX and eculizumab.

A 61‐year‐old woman diagnosed with kappa light‐chain multiple myeloma in September 2009 was initially treated with lenalidomide, bortezomib, and dexamethasone. Upon disease progression in 2010, she received cyclophosphamide, bortezomib, and dexamethasone. In 2011, with worsening in her disease status, the patient underwent high‐dose melphalan followed by autologous hematopoietic stem cell infusion. Post‐transplant, the patient received radiotherapy with 20‐Gy over 10 fractions to the lytic lesions in both hips and femur. She remained in remission until 2014, but with disease relapse, she was retreated with bortezomib and dexamethasone. Unfortunately, her disease status worsened, and she was initiated on newly approved carfilzomib and dexamethasone. She was able to attain a partial response. On day five, cycle nine of this treatment, the patient presented to the hospital with chest discomfort and shortness of air that was progressively worsening over 5 days. On presentation, she had elevated blood pressure, Cr of 5.45 mg/dL (from 1.45 mg/dL), Hb of 7.6 g/dL (from 10.3 g/dL), and Plt count of 37 × 10^9^/L (from 174 × 10^9^/L) along with LDH of 867 IU/L and haptoglobin of <10 mg/dL. PBS was consistent with increased schistocytes. She was emergently started on PLEX; however, her renal function continued to rapidly decline requiring hemodialysis (HD). The patient's underlying multiple myeloma disease status and laboratory work‐up including free kappa light chain, serum‐free lambda light chain, and kappa‐to‐lambda ratio remained unchanged. Upon receiving results of ADAMTS13 activity of 100%, the patient initiated eculizumab therapy. Within 2 weeks of initiating 900 mg IV (weekly) eculizumab, her labs normalized (Fig. 3), and her clinical status significantly improved. She was weaned off HD around 6 weeks (Fig. 4) in the outpatient setting. Patient was maintained on 1200 mg IV eculizumab every 2 weeks from 5th week after initial 900 mg IV every week dosing for 4 weeks in outpatient settings.

## Discussion

**Figure 5:**
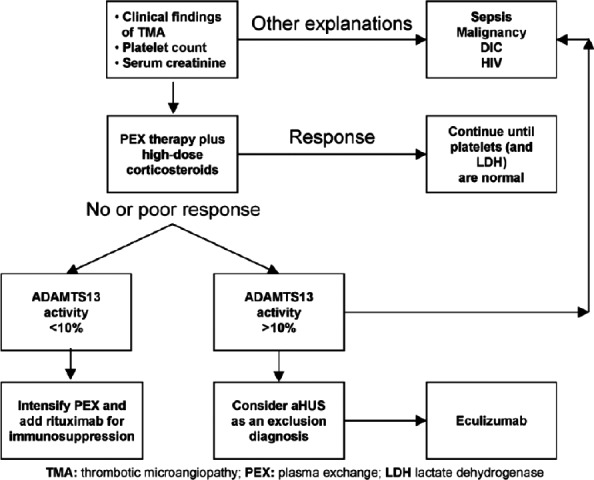
Treatment algorithm for patients presenting with TMA 14.

Atypical HUS is a form of TMA resulting from dysregulation of the alternative complement pathway. The dysregulation triggers thrombotic changes in small blood vessels causing multi‐organ injury, platelet consumption, and fragmentation of red blood cells (RBC). Clinically, this results in hemolysis with schistocytes seen on PBS, thrombocytopenia, and renal failure 11. Etiology of TMA needs to be distinguished because the treatment approach varies based on their underlying pathophysiology mechanism. TTP is a form of TMA driven by ultra‐large molecular weight multimers of von Willebrand factor due to deficiency of the metalloprotease enzyme, ADAMTS13. HUS is a form of TMA propelled by an infection, and aHUS is driven by uncontrolled complement activation. TMA remains important as TTP has a high rate of response to PLEX, whereas aHUS is refractory to PLEX, and eculizumab is the drug of choice when the diagnosis is confirmed 13, 14 (Fig. 5).

Multiple causes including genetic mutations, complement abnormalities, malignancies, pregnancy, organ transplantation, and drugs could induce a pro‐inflammatory state and result in unmonitored activation of the complement system to result in aHUS. This article illustrates an uncommon but a serious complication of two commonly used medications in treating cancer, gemcitabine, and carfilzomib being the underlying cause of aHUS. In the above cases, the patients were actively receiving chemotherapy for their respective malignancies. The incidence of gemcitabine‐induced aHUS is low, but the reported mortality remains as high as 60% 15. It is believed that new onset of hypertension or exacerbation of hypertension is a clue to presence of aHUS, as seen in both of our cases above and many other cases 16. The true incidence of carfilzomib‐induced aHUS remains unknown at this time; however, there have been four case reports prior to our study. One continues to speculate that with increasing use of newer novel agents and physicians being more cognizant of adverse events to these medications, the incidence of drug‐induced aHUS will increase.

With addition of eculizumab, targeting the complement system is changing the schema of aHUS treatment and the prevention of recurrence. The monoclonal antibody that targets the terminal complement protein C5 shuts off downstream generation of cytotoxic membrane attack complex and inflammatory molecules which produce the clinical consequences of aHUS. This has resulted in a high response rate both in terms of hematologic parameters and recovery of renal function, which is one of the most frequent complications of aHUS 1, 2, 13.

## Conclusion

Drug‐induced aHUS is a rare condition; however, early diagnosis is vital as it causes significant morbidity and mortality. With confirmation of the diagnosis, eculizumab appears to be a viable treatment to suppress the pro‐inflammatory surge along with discontinuing the offending agent. Though, the duration of the treatment with eculizumab in this patient population remains unclear. Furthermore, rare adverse reactions of medications such as carfilzomib and gemcitabine should be considered in the appropriate clinical setting.

## Conflicts of Interest

Rahul Gosain M.D., Amitoj Gill M.D., Jacob Fuqua, Lesley H. Volz Pharm.D., Ryan Bycroft Pharm.D., Sarah Seger Pharm.D., Rohit Gosain M.D., Jorge Rios M.D., and Ju‐Hsien Chao D.O.: declare that there is no conflict of interest regarding the publication of this manuscript. Mika R. Kessans Knable Pharm.D.: Speaker Bureau of Alexion.

## Authorship

RG, AG, and JF: conceptualized the original article. RG, AG, JF, LV, MK, RB, SS, RG, JR, and JC: participated in the delivery of patient care and article write‐up.
